# Copy number variation and microdeletions of the Y chromosome linked genes and loci across different categories of Indian infertile males

**DOI:** 10.1038/srep17780

**Published:** 2015-12-07

**Authors:** Anju Kumari, Sandeep Kumar Yadav, Man Mohan Misro, Jamal Ahmad, Sher Ali

**Affiliations:** 1 Molecular Genetics Laboratory, National Institute of Immunology, New Delhi-110067, India; 2 Department of Reproductive Biomedicine, National Institute of Health and Family Welfare, New Delhi-110067, India; 3 Centre for Diabetes and Endocrinology, JN Medical College and Hospital, Aligarh Muslim University, Aligarh, Uttar Pradesh-202002, India

## Abstract

We analyzed 34 azoospermic (AZ), 43 oligospermic (OS), and 40 infertile males with normal spermiogram (INS) together with 55 normal fertile males (NFM) from the Indian population. AZ showed more microdeletions in the *AZFa* and *AZFb* regions whereas oligospermic ones showed more microdeletions in the *AZFc* region. Frequency of the *AZF* partial deletions was higher in males with spermatogenic impairments than in INS. Significantly, *SRY*, *DAZ* and *BPY2* genes showed copy number variation across different categories of the patients and much reduced copies of the DYZ1 repeat arrays compared to that in normal fertile males. Likewise, INS showed microdeletions, sequence and copy number variation of several Y linked genes and loci. In the context of infertility, STS deletions and copy number variations both were statistically significant (p = 0.001). Thus, semen samples used during *in vitro* fertilization (IVF) and assisted reproductive technology (ART) must be assessed for the microdeletions of *AZFa, b* and *c* regions in addition to the affected genes reported herein. Present study is envisaged to be useful for DNA based diagnosis of different categories of the infertile males lending support to genetic counseling to the couples aspiring to avail assisted reproductive technologies.

Human male infertility is caused owing to various factors including genes located on the Y chromosome^1^. The infertility is defined as an inability of a couple to have a child and both male and female factors are equally accountable for^2^. Male infertility is often related to the spermatogenic failure^3^,^4^,^5^. In spite of having normal spermiogram, a significant proportion of the males remain infertile. Based on the World Health Organization (WHO) guidelines^6^, males with spermatogenic impairments may be oligozoospermic (OS) having sperm count <15 million/ml and azoospermic (AZ) where ejaculate is devoid of sperms. Some of the known causes of infertility include testicular pathologies, systemic diseases, endocrinological disorders and obstruction or absence of seminal pathways. Despite these known causes, infertility in about 50% males cannot be explained and such condition is termed as idiopathic infertility^7^. In the context of human male infertility^8^,^9^,^10^, various genes on the human Y chromosome have been implicated besides hundreds of those on the autosomes.

Structurally, human Y chromosome contains pseudo autosomal regions (PARs) and the male specific region of the Y chromosome (MSY) which contains most of the important genes. Owing to the absence of homologous chromosome, Y does not participate in recombination (except PARs) resulting in retention of all the acquired mutations^11^, which are faithfully inherited by the subsequent male generation. The azoospermic factors (*AZF*) i.e. *AZFa*, *AZFb* and *AZFc* and the genes located therein are known to be crucial for the maintenance of normal spermatogenesis^9^,^12^,^13^,^14^. Besides, azoospermic factors, *SRY* gene and DYZ1 arrays are reported to be affected in the blood DNA samples of infertile males having normal spermiogram^15^. Mutations in and copy number variations of the *SRY* gene have been reported earlier in gonadal dysgenesis and sex reversal cases^16^,^17^. Based on literature reports, we hypothesize that the infertile males belonging to different categories would show differences in the frequency and pattern of deletion compared to the normal fertile ones used as control. We analyzed germ line (sperm) DNA samples from OS and infertile males with normal spermiogram (INS) and semen samples from AZ males to gain an insight into the most frequently affected Y linked genes and loci.

## Results and Discussion

Attempts have been made to characterize human Y chromosome in the context of infertility. However, it is still not clear as to how many genes/loci are actually involved in causing reduced level of sperm production or total fertility failure amongst different categories of infertile males. Present study precisely addresses these issues keeping in view the overall significance of the human Y chromosome in human in/fertility.

### MSY Region Mapping using *TSPY-TSPY, AZFa* and *AZFc* candidate STSs

**Figure 1:**
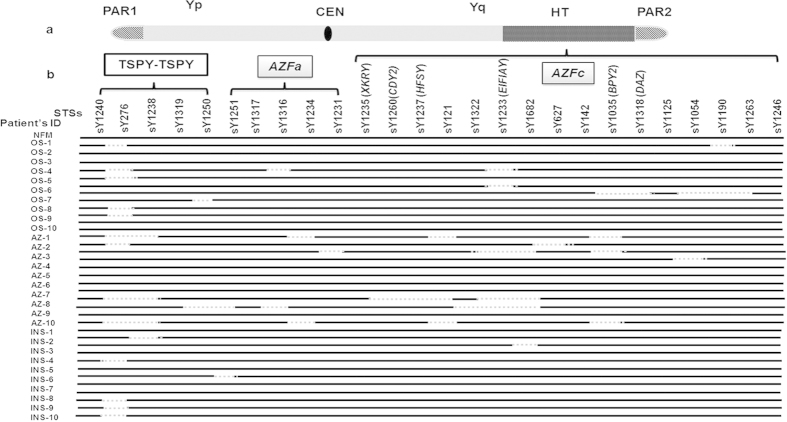
Diagrammatic illustration showing analysis of the MSY region in the patients’ samples. (**a**) Represents human Y chromosome. HT indicates the heterochromatin region of Y chromosome; CEN, centromere and PAR, psuedoautosomal regions. (**b**) Regions on the Y chromosome where STS were analyzed. Sample IDs are given on the left. NFM represent normal fertile males; OS, oligospermic; AZ, Azoospermic and INS, Infertile males with normal spermiogram. Black solid lines for relates to the samples indicating the presence of the STS analyzed, whereas the dotted lines represent deletion of the same.

The landmark STSs from the MSY region of the Y chromosome from different categories of males were screened for different known recombination deletions encompassing P5-Proximal P1, P5-Distal P1, gr/gr, b1/b3, b2/b3, TSPY-TSPY besides checking for the presence of AZFa region (Supplementary table 1). Of these, 14 OS, 11 AZ and 23 INS males showed random microdeletions of *TSPY*-*TSPY* STSs (Fig. 1). Amongst OS patients, sY276 and sY1250 were frequently deleted while AZ and INS showed deletions of sY276 and sY1238. *TSPY* gene is present in multiple copies and distributed on both the arms of the Y chromosome^18^, expressed in testis and spermatogonia, respectively^19^. Copy number variation in this gene was more common in the infertile males compared to the normal controls^20^ and it plays an important role in the context of infertility^21^. Further, at the outset of meiosis, product of this gene has been reported to be involved in signaling process^22^. In the present study, the frequency of STS deletions in the *TSPY-TSPY* region was found to be more common in the INS than in any other categories of the males. This may be due to lack of proper signaling suggesting requirement of its intactness for fertility. Similarly, random microdeletions of candidate STSs in the *AZFa* region were observed in 9 OS, 9 AZ and 10% INS males. These deletions in OS males were specific to sY1316; Azoospermic to sY1234, sY1231, sY1251 and sY1316 and INS to sY1251 STSs. Screening of the landmark STSs from the *AZFc* region showed partial deletions in 14 OS, 18 AZ and 5 INS cases. STSs sY1190, sY1233, sY1035, sY1318, sY1054, sY1190 and sY1263 were found to be deleted in OS, sY1035, sY627, sY1322, sY1233, sY1235, sY1260, sY1237, sY1682, in AZ males and sY1682 in INS patients. Based on these observations, we undertook detailed analyses of the *AZF* regions in these patients.

### Vulnerability of HERV region in the Azoospermic males and microdeletions in the *AZFb* and *c* regions

**Figure 2:**
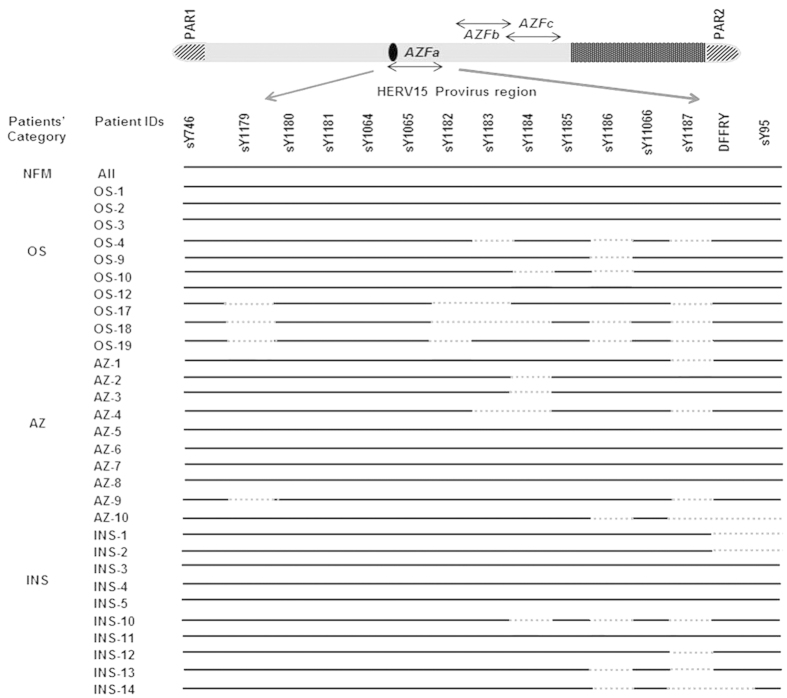
STS mapping of *AZFa* region of the patients. STS mapping of *AZFa* region was done using samples from infertile males of different categories (oligospermic OS, azoospermic AZ, infertile males with normal spermiogram INS) and normal fertile males (NFM). Presence of the corresponding STSs in the patients is indicated by solid lines and absence by dotted lines; patient IDs and their categories are shown on the left. As expected, all the STSs were found to be intact in the fertile male samples used as control.

STSs specific to HERV sequences^4^ in the *AZFa*, *b* and *c* regions were found to be intact in normal fertile males with normal spermiogram (NFMs). Random microdeletions across the *AZFa* region were observed in the patients while none showed known HERV mediated recombination (Fig. 2). Based on the published literature^4^, the first HERV mediated recombination event involved deletion of STSs sY1064, 1065, 1162, 1163, 1164, 1165 and 1166. The second recombination event results in deletion of sY1180, sY1181, sY1064, 1065 and 1162. Deletions in HERV regions were more frequent in the AZ males than that in other categories of the infertile males. About 14 OS, 19 AZ and 10 INS males showed partial deletions of these STSs analyzed. In the infertile males, STSs in the HERV Provirus B region are more affected compared to those in Provirus A region. Interestingly, two INS showed a deletion pattern similar to the second HERV recombination mediated event except sY1064 that remained intact. On the human Y chromosome, *USP9Y* and *DBY* are the two important genes located in between the two HERV provirus regions. We analyzed the STSs specific to *USP9Y* and *DBY* genes. sY1316 was deleted in ~13% OS males. Similarly 4% OS, 9% AZ and 6% INS males showed deletions of sY1317 and sY1234, suggesting the vulnerability of *USP9Y* and *DBY* genes. The deletion frequency of **DBY1** was more prevalent in AZ males (20%) as compared to that in any other categories.

**Figure 3:**
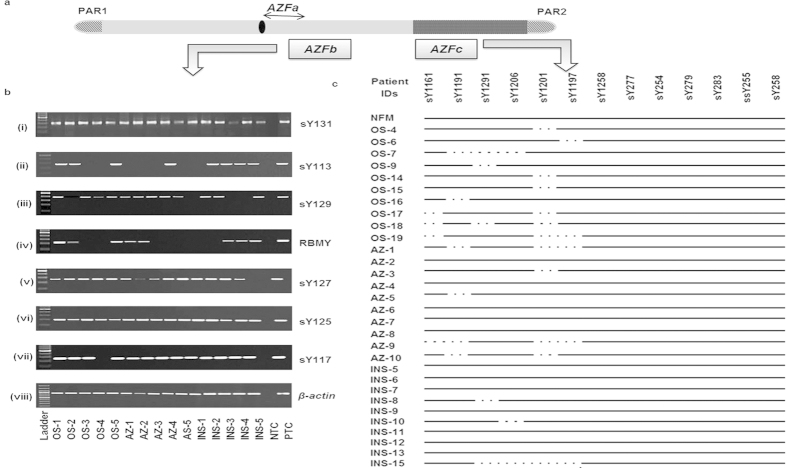
STS mapping of *AZFb* and *AZFc* regions. (**a**) Diagrammatic illustration of human Y chromosome. (**b**) Representative gels (i-viii) showing STS mapping of *AZFb* region for some patients. *β-actin* was used as an internal control. The STSs analyzed are shown on the right side and the patients IDs, in the bottom. NTC and PTC indicate the negative and positive controls, respectively. NFM, OS, AZ and INS indicate normal fertile males, oligospermic, azoospermic and infertile males with normal spermiogram, respectively. (**c**) *AZFc* STS mapping results of some of the representative samples from each category of males. Presence of the corresponding STSs (given on the top) in the patients are indicated by solid line and absence by dotted ones. The patient IDs are shown on the left side.

In the *AZFb* region, STSs sY113, sY117 and sY129 were found to be commonly deleted in all the categories of patients (Fig. 3b). Approximately, 8% OS, 36% AZ and 12% INS patients showed deletion of sY113. Similarly, STS sY117 was found to be deleted amongst 8% OS and 9% AZ males. About 6% INS showed deletion of sY129. The *AZFb* candidate gene *RBMY* specific STS (F19/E355) was found to be deleted in 4% OS, 18% AZ and 6% INS males. In total, approximately, 13 OS, 19 AZ and 3 INS patients showed random partial deletions across the *AZFb* region.

**Figure 4:**
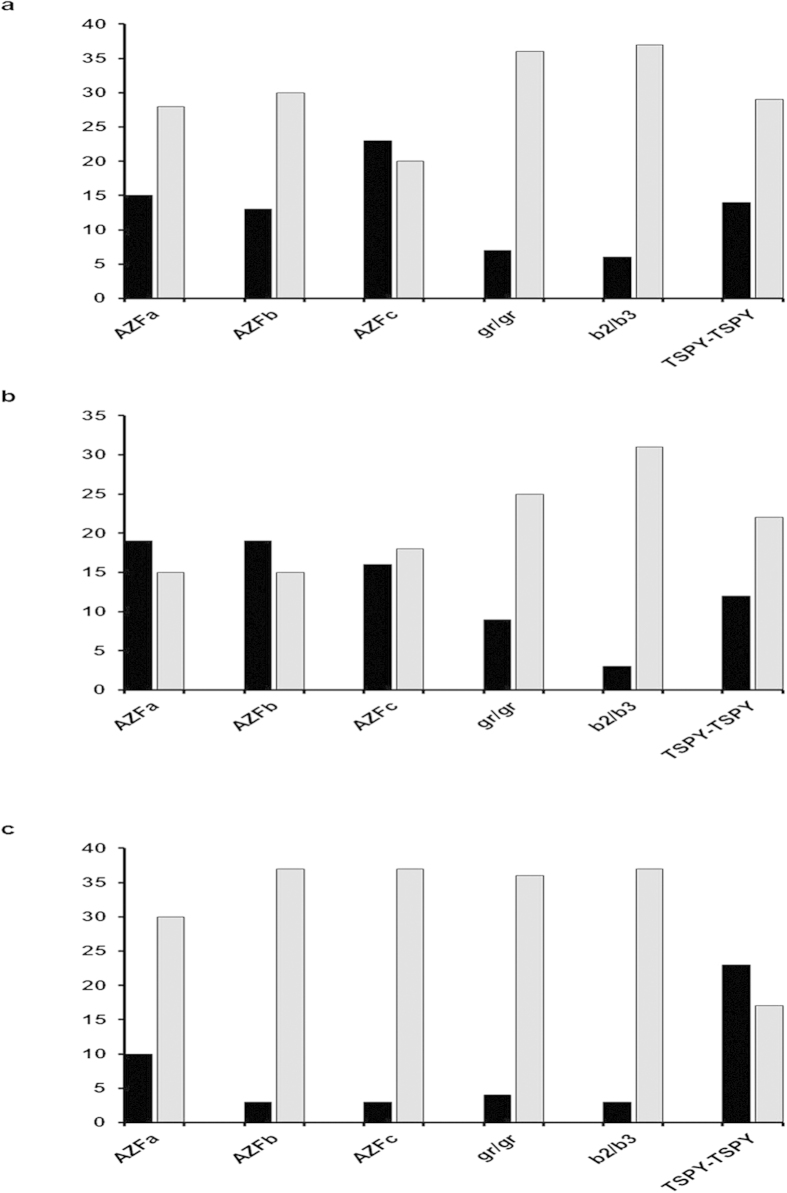
Deletion frequency in the *AZF* regions in the infertile males from different categories. X-axis depicts the deletions in different regions of the Y chromosome, whereas the Y-axis shows % of the patients. Black bars indicate the % of males (belonging to particular category) with deletions and grey bars correspond to % of males without deletions. Panels **a**, **b** and **c** are for the OS, AZ and INS respectively. No deletions were observed in the normal fertile males.

Of all the STSs studied from *AZFc* region, sY1161, sY1191 (b2/b3 deletion), 1291 (gr/gr deletions), sY1197, sY1201 and sY1206 were commonly deleted in these infertile males (Fig. 3c). However, the proportion of partial *AZFc* deletions was higher in OS^23^ and AZ^16^ males than that in INS patients. 7 OS, 9 AZ and 4 INS patients showed gr/gr deletions, whereas 6 OS, 12 AZ and 3 INS patients showed deletion of b2/b3 (sY1191). Among these, two OS patients showed both gr/gr and b2/b3 deletions. The frequency of deletions across the *AZFa*, *b* and *c* regions including gr/gr, b2/b3 and *TSPY-TSPY* are summarized in Fig. 4. However, we didn’t observe complete P5-P1 proximal or distal recombination deletion events which are also considered to be important for spermatogenesis. The Fisher’s exact test confirmed that the *AZFa*, *AZFb*, *AZFc*, gr/gr, b2/b3 and *TSPY-TSPY* deletions in the infertile males of different categories (OS, AZ and INS) were statistically significant (p = 0·001) compared to those in NFMs. Deletions in the *AZFa*^4^, *AZFb*^9^ and *AZFc*^23^ have been reported in males with reduced sperm count. The *AZFa* and *AZFb* regions are considered important for the initiation of spermatogenesis. However, normal spermatogenesis requires intact *AZFc* region^24^. Thus, even partial deletions of gr/gr, b1/b3, b2/b3 and b3/b4 belonging to the *AZFc* region may subvert the process of spermatogenesis. Similarly, *USP9Y* and *DBY* genes have been reported to be crucial for normal spermatogenesis^8^,^25^,^26^ and microdeletions in these genes that we detected correlate well with the infertility status of the males. Human Y chromosome contains eight different palindromic sequences which are comprised of inverted duplicates resulting in local recombination events involving the *AZF* region. These intrachromosomal recombinations lead to microdeletions across the region and sometimes even help in eliminating the deleterious mutations. Presence of these palindromic sequences signifies the possible mechanism through which Y chromosome is protected against degeneration^27^. In the absence of homologous recombination partner for Y chromosome, mutations acquired in one generation are faithfully inherited to the next one.

Partial deletions like gr/gr and b2/b3 have been reported to be associated with an increased risk of complete deletion of the *AZFc* in the subsequent generations^28^. The gr/gr deletions and the corresponding *DAZ* gene copy deletions were found to have impact on the spermatogenesis^29^. A strong association of b2/b3 sub-deletion and male infertility was reported in Chinese population^30^. Microdeletions in different *AZF* regions of the Y chromosome amongst infertile males have been reported to vary across the world populations such as China, Japan, Turkey and USA^31^,^32^,^33^,^34^,^35^,^36^,^37^. These variations may be attributed to the differences in the inclusion and exclusion criteria of patients, methodologies used for study, geographical locations, ethnicity of the study subject and sample sizes. Therefore, a quest for consensus on genotypic changes across the different categories of patients, though highly desirable, will continue to remain a challenge.

### SNV Analysis of *DAZ* and *BPY2* genes

SNV analyses of these two genes across different categories of the infertile males showed differences in the restriction digestion pattern (Supplementary Figure S1 and Table 1). The STS sY152 was found to be deleted in twenty patients (7 OS, 9 AZ and 4 INS), whereas STS Y-DAZ3 showed deletion in twenty one (6 OS, 12 AZ and 3 INS) patients. *DAZ* was found to be present in the form of four copies in two clusters in the *AZFc* region of the Y chromosome^38^. The importance of the *DAZ* gene clusters in the spermatogenesis is well known^39^,^40^,^41^. *DAZ1/DAZ2* and *DAZ3/DAZ4* deletions were reported in the infertile men but not in normal fertile males^39^. Thus, deletion of *DAZ1/DAZ2* was associated with spermatogenic impairment, whereas loss of *DAZ3/DAZ4* had little or no effect. Here, we observed the gr/gr and associated *DAZ* deletions even in INS suggesting that the *DAZ* gene deletions are not sufficient to impair the spermatogenesis. A similar report from China suggests that *DAZ* cluster deletions may not be sufficient to result in spermatogenic impairment^42^. Similarly, SNV analysis of *BPY2* gene showed about thirty nine (11 OS, 21 AZ and 7 INS) patients having allele A only, whereas others had both A and B alleles. The patient samples with only one of the alleles sequenced, showed single nucleotide variations. In all the NFMs, both the alleles of *DAZ* and *BPY2* genes remained intact. Also, our work provides evidence *albeit* indirect that these deletions are independent of endocrinological control. This is more suggestive from the case analysis of INS.

In the present study, AZ, OS and INS, genes specific STSs showed deletions. Previously, these deletions were linked to the infertile males having less sperm counts compared to the normal ones suggesting that they are important for spermatogenesis. However, in the present study, deletions in the INS males and in some cases reported in normal fertile controls indicate that only such deletions might not be responsible for reduced sperm count. As the deletion frequencies of these genes were higher in AZ males compared to other categories of infertile males, one can argue that the dose effect of these two genes (*BPY2* and *DAZ*) and structural integrity of Y chromosome in general are important for deciding the fate of spermatogenesis. However, increased dosage of the genes could also be deleterious in context of the male infertility. Ye *et al.*, 2013^43^ have shown that partial *AZFc* duplications, not the gr/gr or b2/b3 deletions were associated with the male infertility in Chinese-Yi population.

### DYZ1 arrays showed copy number variations

**Figure 5:**
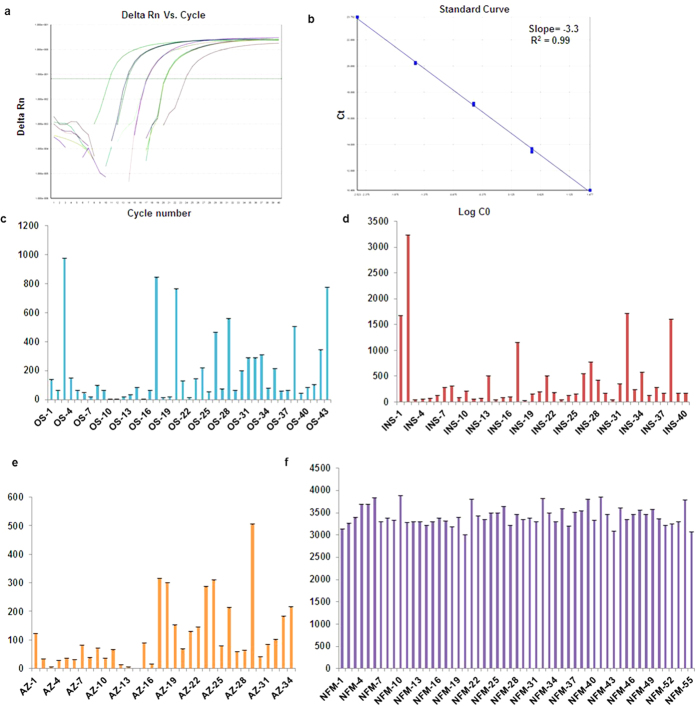
Copy number analysis of DYZ1 arrays by qPCR. Representative amplification plot (**a**) and standard curve (**b**) used for the copy number calculation of DYZ1 arrays. The bar graphs (**c–f**) show the distribution of copies in the males of different categories including oligospermic (43), infertile males with normal spermiogram (40), azoospermic (34)and normal fertile males (55) respectively. All the reactions were performed in triplicates and the results shown here are average of these triplicates.

The copies of DYZ1 arrays calculated using quantitative Real Time PCR (qPCR) were found to be significantly reduced in the infertile males (Fig. 5). Statistical analysis of DYZ1 copies conducted by using one way ANOVA test in infertile males and normal fertile males showed normal distribution on the log scale. Copy number variations of the DYZ1 arrays among the infertile males were statistically significant (p = 0·0001) compared to that in NFMs. Upon applying the Bonferroni adjustment, copy number differences between the AZ and NM (p = 0·001); OS and NM (p = 0·001); INS and NM (p = 0·001); INS and AZ (p = 0·001); INS and OS (p = 0·004) were found to be statistically significant. However, the same was non-significant in the OS and AZ (p = 1·000) patients. Thus, reduced copies of DYZ1 arrays in the infertile males were substantiated by statistical analysis which was not reported earlier. Significantly, reduced copies of DYZ1 have been reported in prostate cancer, repeated abortion cases, males exposed to natural background radiation and ground water arsenic^44^,^45^. Taken together, we hypothesize that the DYZ1 arrays in adequate number are necessary for the maintenance of structural and functional integrities of the Y chromosome.

**Figure 6:**
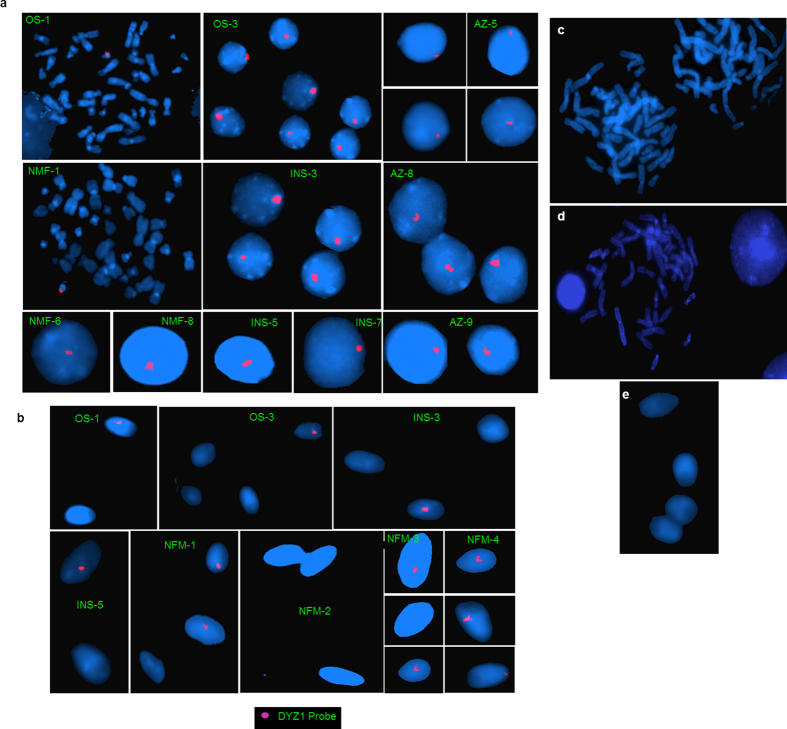
Localization of DYZ1 on metaphase chromosomes, interphase nuclei and spermatozoa using FISH. (**a**) DYZ1 probe was labeled with Texas red, whereas metaphases and interphase nuclei were stained with DAPI. (**b**) Sperm nuclei are stained with DAPI. The Y bearing sperms are showing red signal of DYZ1, whereas the X bearing sperms lack the DYZ1 signal. (**c**) Male blood metaphase and (**e**) sperm samples were processed together with the experimental samples under identical conditions but not hybridized with the DYZ1 probe to exclude the background signal. (**d**) Female sample hybridized with DYZ1 probe. Patient IDs are shown in green. OS indicates oligospermic; AZ, azoospermic; INS, infertile males with normal spermiogram and NFM, normal fertile males.

DYZ1 was localized onto the metaphase chromosome, interphase nuclei and spermatozoa (Fig. 6). The intensity of the signal varied amongst the infertile patients suggesting their possible copy number variation. The semen samples contain either X- or Y- bearing sperms. The probe localized the DYZ1 onto the Y- bearing spermatozoa with red signal, while the X-bearing spermatozoa, as expected, lacked the signals.

### Copy Number Status of *DAZ* and *BPY2* Genes using TaqMan Assays

**Figure 7:**
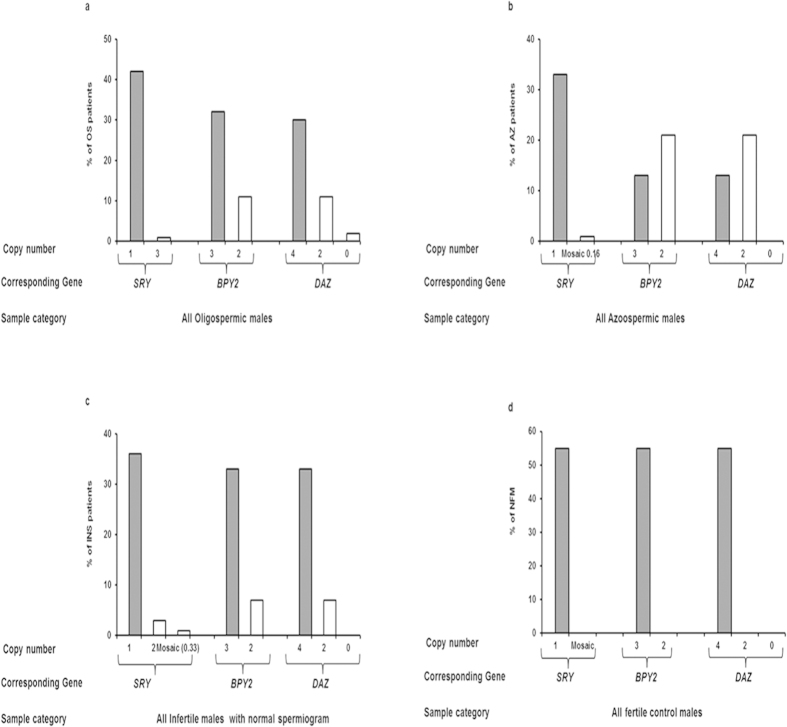
Copy number estimation of *SRY*, *BPY2* and *DAZ* genes using real time PCR. (**a**–**d**) Summarizes the distribution of copy number of *SRY*, *BPY2* and *DAZ* in oligospermic (**a**), Azoospermic (**b**), infertile males with normal spermiogram (**c**) and normal fertile males (**d**), respectively. X-axis depicts the gene of interest analyzed and the copy number assessed using real time PCR while the Y-axis indicates the % of males with corresponding copies for the genes. Grey bars indicate the normal copy number for the particular gene and the white bars indicate the copy number variation observed. A total of 43 OS, 34 AZ and 40 INS patients were analyzed for copy numbers and each reaction was set in triplicates during real time PCR amplification.

The copy number analyses of *BPY2* and *DAZ* genes showed variation amongst the infertile males. About 11 OS, 21 AZ and 7 INS patients showed deletion of two copies of *DAZ* and one copy of *BPY2*. Two OS males showed deletion of all the four *DAZ* copies, while the remaining patients had all the four copies *of DAZ* gene. The frequency of copy number variations (number of patients having corresponding *DAZ/BPY2* copies) across the OS, AZ and INS patients are shown in Fig. 7a, b and c (Supplementary Figures 2 and 3). The results of copy number analyses of *DAZ* and *BPY2* genes were found to be in accordance with the data of our SNV analysis. Statistically, deletions of the *BPY2* and *DAZ* genes were found to be significant (p = 0·001) in the infertile males belonging to all the categories based on Pearson chi square exact test. These association should be taken into consideration with due caution because studies on this line have shown inconsistent and at times controversial results.

### Copy number polymorphism of the *SRY* gene

**Figure 8:**
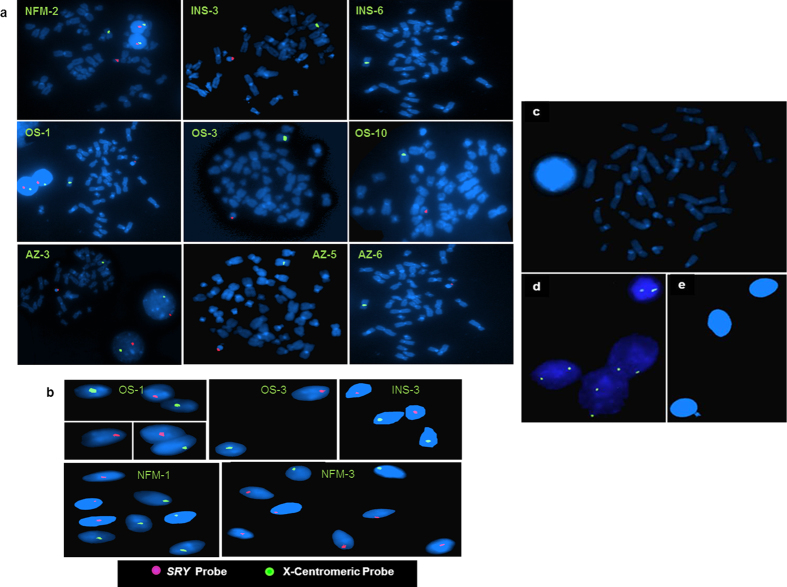
Chromosomal localization of the SRY gene on metaphases, interphase nuclei and spermatozoa using Fluorescence *in situ* hybridization (FISH). (**a**) The metaphase and interphase nuclei are stained with DAPI. The *SRY* gene localized on the Y chromosome is showing red signal and X-centromere, fluorescent green signal. (**b**) Figure represents mapping of *SRY* gene on the individual sperm. All the Y bearing sperms are shown in red, whereas those of X-bearing ones are shown in green. (**c**) Male blood metaphase and (**e**) sperm samples were processed under identical conditions but not hybridized with the *SRY* probe to exclude the background signal. (**d**) Female sample hybridized with *SRY* probe. The spermatozoa are stained with DAPI. Patient IDs are shown in yellow. AZ indicates azoospermic; OS, oligospermic; INS, infertile males with normal spermiogram and NFM, normal fertile male.

The intactness of *SRY* gene was confirmed using PCR (Supplementary Figure 4). Copy number estimation showed that all NFMs had one copy. All OS and AZ males (Fig. 7a,b) had one copy of *SRY* (normal) except two patients (one OS and one AZ) who had 3 copies and less than one copy (0·33) of the *SRY*, respectively, suggesting mosaicism of the same in later. Among the INS, thirty six males showed normal *SRY* copies (Fig. 7c and Supplementary Figure 2), while three showed two and one male had less than one copy (0·36, based on real time PCR data) of the *SRY* gene. In summary, 95% of the patients showed normal one copy of the *SRY* gene, while the others showed either loss or gain of the same. *SRY* gene was localized onto the chromosomal metaphase and spermatozoa (Fig. 8). In most mammals, including humans, testis determination is regulated by *SRY* gene leading to male differentiation^46^. It is equally likely that some of the sperms may be lacking normal *SRY* gene resulting in detection of less than one copy by Real Time PCR. Copy number variation in the *SRY* gene has been reported in males exposed to natural background radiations, patients suffering from sex chromosome related anomalies and Turner Syndrome^47^,^48^ and now in a few infertile males.

#### Conclusions

The main focus of this study has been to uncover consensus, if any, amongst different categories of infertile males. We uncovered inconsistency in STS deletion, CNV and mutational load amongst the males belonging to a given clinical category. Present study establishes a correlation between frequent aberrations of several Y chromosome linked genes and loci including DYZ1 region with various categories of infertile males. Owing to polygenic conditions and absence on genotypic consensus, development of biomarker for unequivocal DNA based diagnosis would continue to remain a challenge. Nonetheless, based on the present results, we suggest semen samples used for IVF and ART must be evaluated for the intactness of vital STSs and the candidate genes and loci.

## Methods

All the methods were carried out in accordance with the approved guidelines of the National Institute of Immunology, New Delhi and all experiments were carried out in accordance with the approved guidelines.

### Subjects

The germ line samples were collected from the Department of Endocrinology, J.N. Medical College, Aligarh Muslim University, Aligarh. We included couples who were unable to have children after attempting for more than one year. Both the partners of each couple were clinically examined and only those males were recruited for the study whose female partners were clinically normal (ovulation, hormonal tests-FSH, estrogen and progesterone, tubal and uterine assessments). The males having any chromosomal abnormalities (karyotyping), congenital absence of vas deferens, known infertility caused by cystic fibrosis, testicular tumors or undergoing chemotherapy and radiotherapy were excluded from the present study. The semen samples were collected from the patients as routine diagnostic practice followed at the clinic and assessed for spermiogram in accordance with WHO (2010) criteria. The samples in excess of the diagnostic requirements were used for DNA analysis.

Samples were collected from infertile males belonging to 43 OS, 34 AZ and 40 INS categories. For positive control, fifty five semen samples from NFMs were collected. Genomic DNA from female blood sample and a reaction without template were included as negative control.

### Ethical approval

The study was approved both, by the Institutional Human Ethical Committee of the National Institute of Immunology, New Delhi and the Bio-Ethical Committee, Aligarh Muslim University, Aligarh. The patients were counseled and apprised about the study and its protocol before obtaining their informed consent.

### DNA isolation

For DNA isolation, semen samples were allowed to liquefy at 37 °C for 20 minutes. These samples were then processed to retrieve sperms using density percoll gradient (80% and 40%) to exclude somatic cells contamination following standard protocols^6^. Samples were re-suspended in 5 ml sperm wash buffer (0·15 mM NaCl, 10 mM EDTA pH 8·0), mixed gently and centrifuged at 2000 g for 10 minutes at 4 °C. This process was repeated twice; pellet was collected and washed twice to obtain a clear supernatant. The pellet was dissolved in sperm lysis buffer (0·1% SDS, 0·5% Triton X-100) and centrifuged at 2000 g for 10 minutes at 4 °C. DNA was extracted from the pellet of spermatozoa using TRI Reagent^®^RT following manufacturer’s instructions (Sigma Aldrich, USA). An equal volume of TRI reagent was added (initial semen sample: TRI reagent), mixed properly and allowed to stand for 10 minutes at room temperature. About 1/10^th^ volume of chloroform was added and mixed. The mixture was centrifuged at 12000 g for 15 minutes at 4 °C. The upper aqueous layer containing RNA was removed. The interphase layer containing DNA was transferred to a fresh tube. About 100 μl of ethanol was added and allowed to stand at room temperature for 10 minutes. The samples were centrifuged at 12000 g for 10 minutes at 4 °C. The pellet was washed with pre-chilled 75% ethanol, air-dried and dissolved in TE buffer. The quantity and quality of DNA was checked by PCR amplification using *GAPDH*, *β-actin*, PRM1 and PRM2 primers (Supplementary table S2). Along with the samples, commercially purchased male blood DNA was used as a positive control for these PCR reactions. Azoospermic patients do not contain detectable sperms in their ejaculates. These samples were processed directly for isolation of DNA following standard protocols^49^.

### Sequence tagged site (STS) mapping of the *AZF* region

*AZF* region of Y-chromosome was analyzed for micro-deletion of STSs using end point PCR amplification^15^. A total of 64 STSs were screened from the *AZFa, b*, and *c* regions in the patients and control samples (Supplementary table S1 and S3).

### Single nucleotide variant (SNV) analyses

For SNV analyses of *DAZ* and *BPY2* genes, PCR-restriction fragment length polymorphism approach was used (Supplementary table S4), employing established protocols^15^.

### Mutational and Copy number variation (CNV) Analyses of *SRY*, *DAZ*, *BPY2* genes and DYZ1 arrays

Mutations in the *SRY* gene were assessed in the infertile patients belonging to different categories through PCR using three pairs of *SRY* specific primers (SRY1, SRY2, and sY14; see details in Table 1). The copy numbers of the DYZ1 arrays of the patients were calculated by qPCR based on absolute quantification method using SYBR green (Life technologies, California, USA). Copy numbers of the *SRY*, *DAZ*, and *BPY2* genes were calculated using TaqMan probes for the respective genes following standard protocols^15^.

### Metaphase preparation, DYZ1 probe labeling and Fluorescence *in-situ* Hybridization

Metaphase chromosomes were prepared from the blood samples following standard protocols for FISH^50^. Likewise, sperm samples were processed for conducting FISH experiment. An aliquot of semen samples were washed in 1X PBS, centrifuged at 300 g for 5 minutes. The process was repeated twice and the sperms were then fixed in freshly prepared and chilled fixative. The sperm samples were stored at −20 °C until used for FISH. The DYZ1 plasmid was nick translated with Texas red-12-dUTP (Invitrogen, USA) using nick translation kit (Vysis, USA) following supplier’s specifications. The nick translated probe was used for hybridization of the metaphases and spermatozoa samples. FISH probe “LSI SRY Spectrum Orange/CEP X Spectrum Green” was procured from Vysis (Illinois, USA). This probe can localize simultaneously the *SRY* gene on the Y and centromeric sequences on the X chromosome. The FISH was conducted on the spermatozoa following standard protocol^51^ with some modifications. The labeled FISH probes, DYZ1 and *SRY* were hybridized with spermatozoal and metaphase spread samples. The images were captured using fluorescent microscope BX51 equipped with TV1X-2 camera (Olympus, Japan) and CytoVision-Genus^TM^ imaging software (Applied Imaging, USA). Each slide was screened for about 200 non overlapping nuclei and metaphases. To exclude the chances of background signal, male blood metaphase and sperm samples were processed together under identical conditions but were not hybridized with the respective probes. As control, female metaphase samples were hybridized with DYZ1 and *SRY* probes.

### Statistical analysis

Statistical analysis was performed using Stata IC/12.1. The differences amongst the different groups of patients and fertile males in the context of *AZFa*, *AZFb*, *AZFc*, gr/gr, b2/b3, and *TSPY-TSPY* deletions were tested using Fisher’s exact test. Similarly, the copy number variations in *BPY2* and *DAZ* genes were tested using the Pearson chi square exact test. The copy number variations of DYZ1 arrays in different groups of infertile males and fertile control ones were analyzed using One Way Analysis of Variance (ANOVA) and a Bonferroni adjustment was done for the multiple comparison test (pair wise comparison and post hoc analysis). A p-value less than 0.05 was considered statistically significant for each test.

## Additional Information

**How to cite this article**: Kumari, A. *et al.* Copy number variation and microdeletions of the Y chromosome linked genes and loci across different categories of Indian infertile males. *Sci. Rep.* **5**, 17780; doi: 10.1038/srep17780 (2015).

## Supplementary Material

Supplementary Information

## Figures and Tables

**Table 1:** Summary of SNV analysis in spermatozoal samples across different categories of the patients.

S. No.	SNV type	Patients (%) with allele A + B				Patients (%) with allele A only				Mutations observed in RS	Patients (%) with allele B only				Mutations observed in RS
		OS	AZ	INS	NFM	OS	AZ	INS	NFM		OS	AZ	INS	NFM	
1	*DAZ*-SNV_I	100	100	100	100	–	–	–	–	None	–	–	–	–	None
2	*DAZ*-SNV_II	84	74	90	100	–	–	–	–	None	16	26	10	–	G > A (*MboI*)
3	*DAZ*-SNV_III (sY586)	84	74	90	100	–	–	–	–	None	16	26	10	–	T > C (*TaqI*)
4	*DAZ*-SNV_IV	84	74	90	100	–	–	–	–	None	16	26	10	–	T > C (*AluI*)
5	*DAZ*-SNV_V (sY587)	74	39	55	100	–	–	–	–	None	26	61	45	–	T > C (*DraI*)
6	*DAZ*-SNV_VI	86	65	92	100	14	35	8	–	G > T (*AflIII*)	–	–	–	–	None
7	*DAZ*-SNV_VII (sY581)	100	100	100	100	–	–	–	–	None	–	–	–	–	None
8	*BPY2*	74	39	92	100	26	61	8	–	G > A (*EcoRV*)	–	–	–	–	None

^*^OS, AZ, INS and NFM indicate oligospermic, azoospermic, infertile males with normal spermiogram and normal fertile males, respectively. RS means restriction site specific to a particular SNV, whereas “-”sign indicates absence of the allele. We used 55 control fertile males and all were found to be normal.
