# Supplementary material for: Copy number variation and microdeletions of the Y chromosome linked genes and loci across different categories of Indian infertile males
Source: Sci Rep. 2015 Dec 7;5:17780. doi: 10.1038/srep17780 (PMC4671023; doi:10.1038/srep17780)
Supplement: Supplementary Information [file srep17780-s1.pdf]

Copy number variation and microdeletions of the Y chromosome linked genes and loci across different categories of Indian infertile males

Anju Kumari[1], Sandeep Kumar Yadav[1], M.M. Misro[2], Jamal Ahmad[3], Sher Ali[1*]

[1]Molecular Genetics Laboratory, National Institute of Immunology, New Delhi-110067, India

[2] Department of Reproductive Biomedicine, National Institute of Health and Family Welfare, New Delhi-110067, India.

[3]Centre for Diabetes and Endocrinology, JN Medical College and Hospital, Aligarh Muslim University, Aligarh, Uttar Pradesh-202002, India

Author affiliations

Corresponding author

Dr. Sher Ali

Email: alisher@nii.ac.in; sheralib5@hotmail.com

Molecular Genetics Laboratory

National Institute of Immunology

New Delhi-110067, India

Ph. 91-011-26703753

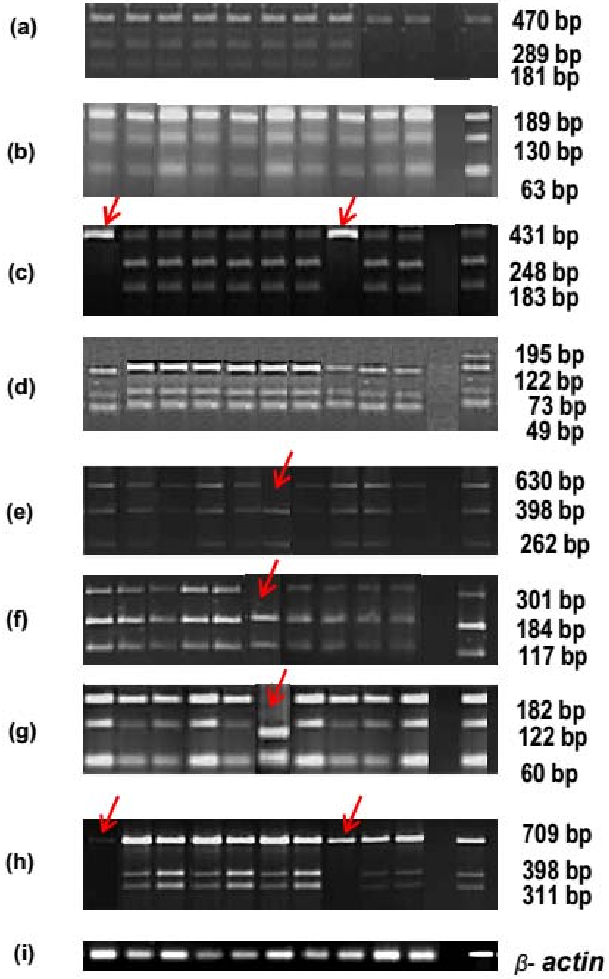

Supplementary Fig. S1. SNV analysis of *DAZ* and *BPY2* genes. **(A) – (H)** show representative gels for *BPY2*_SNV*, DAZ* SNV_I, II, III, IV, V, VI, VII and *β- actin*, respectively. The restriction digestion fragment sizes for the corresponding SNVs are shown on the right side. *β- actin* was used as an internal positive control.

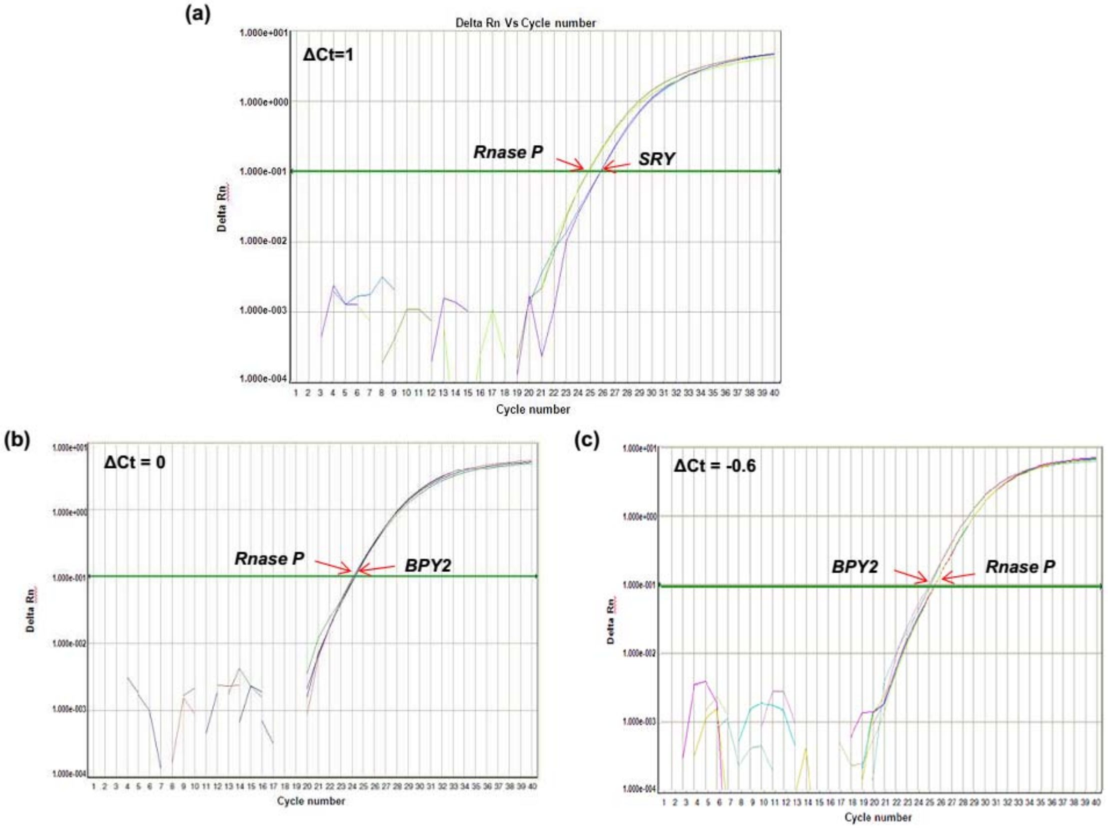

Supplementary Fig.S2 . Amplification plots used for copy number calculation of *SRY* and *BPY2* genes. (A), (B) and (C) are the representative real time PCR amplification plots for copy number estimation of *SRY* and *BPY2* genes. *RNase P* was used as reference gene. ΔCt= 1 represents one copy of SRY gene, similarly ΔCt= 0.6 and ΔCt= 0 correspond to three and two copies of the BPY2 gene, respectively.

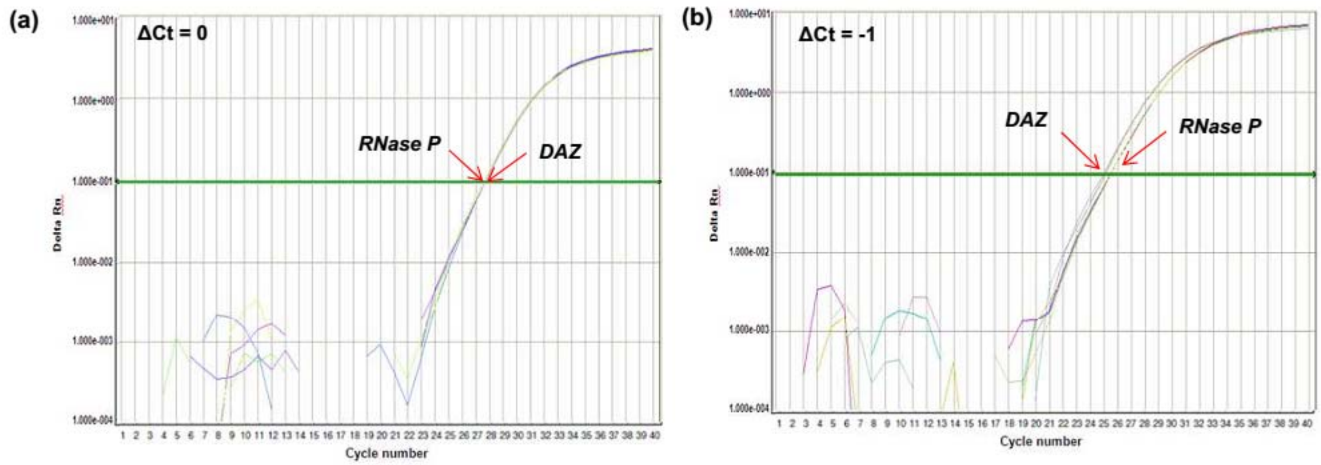

Supplementary Fig. S3. Amplification plots used for copy number calculation of *DAZ* gene. (A) and (B) are representative qPCR amplification plots for *DAZ* gene. ΔCt= 0 and ΔCt= -1 correspond to two and four copies of the *DAZ* genes, respectively.

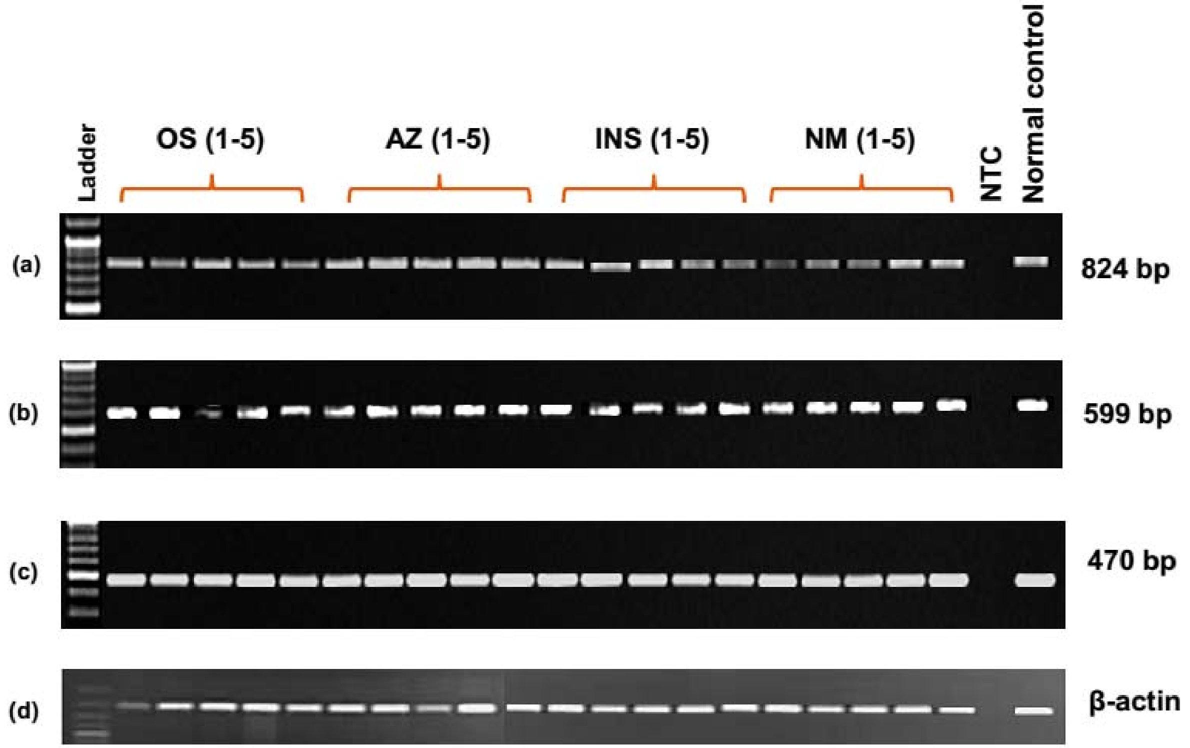

Supplementary Fig. S4 . Intactness of *SRY* gene in germ line samples of patients. The *SRY* gene was assessed for its intactness using primers SRY1 **(A)**, SRY2 **(B)** and sY14 **(C)**. *β*- actin was used as an internal positive control **(D)**. The size of amplicons are shown along the right side. The patient IDs are shown on the top. NTC indicates the negative controls without template DNA. Sample of normal fertile male used as control are given on the right. Here, OS, AZ and INS denote oligospermic, azoospermic and infertile males with normal spermiogram, respectively.

Supplementary Table S1 List of the STSs used for screening of the *AZF*a, *b* and *c* regions

S. No.	*AZF* region	STSs used
1	*AZF*a	sY95, sY746, sY1064, sY1065, sY1066, sY1179, sY1180, sY1181, sY1182, sY1183, sY1184, sY1185, sY1186, sY123, sY1234, sY1251, sY1316, sY1317
2	*AZF*b	sY117, sY125, sY127, sY129, sY113, sY131, sY627
3	*AZF*c	sY121, sY142, sY254, sY255, sY278, sY277, sY1054, sY1125, sY1161, sY1190, sY1191, sY1197, sY1201, sY1206, sY1246, sY1258, sY1263, sY1291, sY1322, sY1682
4	Gene specific STSs	*DBY1*, *DBY2*, F19/E355, sY1035, sY1235, sY1233, sY1237, sY1260, sY1318, sY276, sY1238, sY1240, sY1250 and sY1319, *ZFY*, sY14, RRM3, Y-DAZ3, sY152

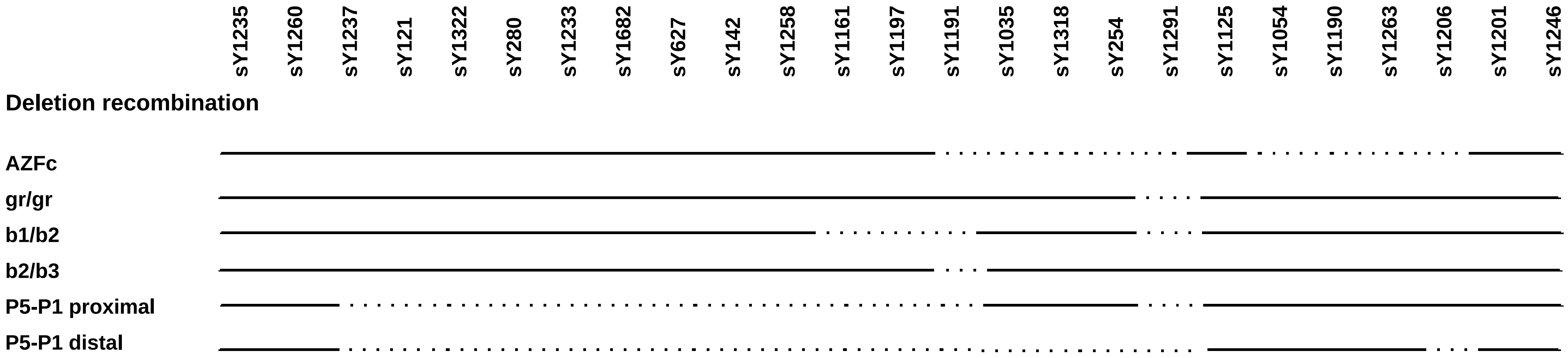

Supplementary table S2 Primers used for end point PCR

Primer	Sequence
β-actin	F- AGATGACCCAGATCATGTTTGAGA<br>R-CTAAGTCATAGTCCGCCTAGAAGC
GAPDH	F- GCCACATCGCTCAGACACCAT<br>R- ACCAGGCGCCCAATACG
***SRY*1**	F- GAATCTGGTAGAAGTGAGTTTTGGA<br>R- GCCTTTATTAGCCAGAGAAAAGAAA
***SRY*2**	F- CTTCTGCTATGTTAAGCGTATTCAA<br>R- CAGCTTTGTCCAGTGGCTGTA
**sY14**	F- GAATATTCCCGCTCTCCGGA<br>R- GCTGGTGCTCCATTCTTGAG

Supplementary Table S3. Details of sizes of different STSs

UniSTS	Size of STS (bp)
sY95	303
sY746	216
sY1064	110
sY1065	239
sY1066	131
sY1179	207
sY1180	200
sY1181	263
sY1182	247
sY1183	201
sY1184	213
sY1185	190
sY1186	179
sY1231	292
sY1234	350
sY1251	422
sY1316	463
sY1317	487
sY113	304
sY117	502
sY125	470
sY127	386
sY129	205
sY131	143
sY627	104
sY121	190
sY142	196
sY254	380
sY255	123
sY277	275
sY1054	340
sY1125	283
sY1161	330
[illegible]	[illegible]

sY1263	467
sY1291	527
sY1322	530
sY1682	437
DBY1	277
DBY2	689
sY1035	348
sY1235	200
sY1233	423
sY1237	150
sY1260	491
sY1318	531
sY276	216
sY1238	375
sY1240	385
sY1250	493
sY1319	303
ZFY	358
sY14	470
RRM3	474
Y-DAZ3	256
sY152	125

Supplementary Table S4 Details of SNVs analyzed for *DAZ* and *BPY2* genes

Gene Target	SNV	Accession no.	Product size (bp)	Restriction enzyme	Restriction site	Fragments Size (bp)	Alleles	Copies
***DAZ* gene**	*DAZ*-SNV_I	*G73167*	709	*FspI*	TGC/GCA	709<br>398+311	A<br>B	1,2,3<br>4
	DAZ-SNV_II	*G73166*	182	*MboI*	/GATC	182<br>122+60	A<br>B	1<br>2,3,4
	sY586	*G63907*	301	*TaqI*	T/CGA	301<br>184+117	A<br>B	2<br>1,3,4
	DAZ-SNV_IV	*G73168*	630	*AluI*	AG/CT	630<br>398+262	A<br>B	2<br>1,3,4
	sY587	*G63908*	244	*DraI*	TTT/AAA	195+49<br>122+73+49	A<br>B	3,4<br>1,2
	DAZ-SNV_VI	*G73169*	431	*AflIII*	A/CRYGT	431<br>248+183	A<br>B	1,2,3<br>4
	sY581	*G63906*	252	*Sau3AI*	/GATC	189+63<br>130+63+59	A<br>B	1,4<br>2,3
BPY2	*BPY2*	*BV012732*	470	*EcoRV*	GAT/ATC	470<br>289+181	A<br>B	2 Copy<br>1 Copy

LEGENDS TO FIGURES

Figure 1. Diagrammatic illustration showing analysis of the MSY region in the patients' samples. (a) Represents human Y chromosome. HT indicate the heterochromatin region of Y chromosome; CEN, centromere and PAR, psuedoautosomal regions. (b) Regions on the Y chromosome where STS were analyzed. Sample IDs are given on the left. NFM represent normal fertile males; OS, oligospermic; AZ, Azoospermic and INS, Infertile males with normal spermiogram. Black solid lines relates to the samples indicating the presence of the STS analyzed, whereas the dotted lines represent deletion of the same.

Figure 2. STS mapping of *AZFa* region of the patients. STS mapping of *AZFa* region was done using samples from infertile males of different categories (oligospermic OS, azoospermic AZ, infertile males with normal spermiogram INS) and normal fertile males (NFM). Presence of the corresponding STSs in the patients is indicated by solid line and absence by dotted lines; patient IDs and their categories are shown on the left. As expected, all the STSs were found to be intact in the fertile male samples used as control.

Figure 3. STS mapping of *AZFb* and *AZFc* regions. (a) Diagrammatic illustration of human Y chromosome. (b) Representative gels (i-viii) showing STS mapping of *AZFb* region for some patients. *β-actin* was used as an internal control. The STSs analyzed are shown on the right side and the patients IDs, in the bottom. NTC and PTC indicate the negative and positive controls, respectively. NFM, OS, AZ and INS indicate normal fertile males, oligospermic, azoospermic and infertile males with normal spermiogram, respectively. (c) *AZFc* STS mapping results of some of the representative samples from each category of males. Presence of the corresponding STSs (given on the top) in the patients are indicated by solid line and absence by dotted ones. The patient IDs are shown on the left side.

Figure 4. Deletion frequency in the *AZF* regions in the infertile males from different categories. X-axis depicts the deletions in different regions of the Y chromosome, whereas the Y-axis shows % of the patients. Black bars indicate the % of males (belonging to particular category) with deletions and grey bars correspond to % of males without deletions. Panels a, b and c are for the OS, AZ and INS respectively. No deletions were observed in the normal fertile males.

Figure 5. Copy number analysis of DYZ1 arrays by qPCR. Representative amplification plot (a) and standard curve (b) used for the copy number calculation of DYZ1 arrays. The bar graphs (c-f) show the distribution of copies in the males of different categories including oligospermic (43), infertile males with normal spermiogram (40), azoospermic (34) and normal fertile males (55) respectively. All the reactions were performed in triplicates and the results shown here are average of these triplicates.

Figure 6. Localization of DYZ1 on metaphase chromosomes, interphase nuclei and spermatozoa using FISH. (a) DYZ1 probe was labeled with Texas red, whereas metaphases and interphase nuclei were stained with DAPI. (b) Sperm nuclei are stained with DAPI. The Y bearing sperms are showing red signal of DYZ1, whereas the X bearing sperms lack the DYZ1 signal. (c) Male blood metaphase and (e) sperm samples were processed together with the experimental samples under identical conditions but not hybridized with the DYZ1 probe to exclude the background signal. (d) Female sample hybridized with DYZ1 probe. Patient IDs are shown in green. OS indicates oligospermic; AZ, azoospermic; INS, infertile males with normal spermiogram and NFM, normal fertile males.

Figure 7. Copy number estimation of *SRY, BPY2* and *DAZ* genes using real time PCR. (a-d) Summarizes the distribution of copy number of *SRY*, *BPY2* and *DAZ* in oligospermic (a), Azoospermic (b), infertile males with normal spermiogram (c) and normal fertile males (d), respectively. X-axis depicts the gene of interest analyzed and the copy number assessed using real time PCR while the Y-axis indicates the % of males with corresponding copies for the genes. Grey bars indicate the normal copy number for the particular gene and the white bars indicate the copy number variation observed. A total of 43 OS, 34 AZ and 40 INS patients were analyzed for copy numbers and each reaction was set in triplicates during real time PCR amplification.

Figure 8. Chromosomal localization of *SRY* gene on metaphases, interphase nuclei and spermatozoa using Fluorescence *in situ* hybridization (FISH). (a) The metaphase and interphase nuclei are stained with DAPI. The *SRY* gene localized on the Y chromosome is showing red signal and X-centromere, fluorescent green signal. (b) Figure represents mapping of *SRY* gene on the individual sperm. All the Y bearing sperms are shown in red, whereas those of X-bearing ones are shown in green. (c) Male blood metaphase and (e) sperm samples were processed under identical conditions but not hybridized with the *SRY* probe to exclude the background signal. (d) Female sample hybridized with *SRY* probe. The spermatozoa are stained with DAPI. Patient IDs are shown in yellow. AZ indicates azoospermic; OS, oligospermic; INS, infertile males with normal spermiogram and NFM, normal fertile male.
